# A global reference for caesarean section rates (C‐Model): a multicountry cross‐sectional study

**DOI:** 10.1111/1471-0528.13509

**Published:** 2015-08-10

**Authors:** JP Souza, AP Betran, A Dumont, B de Mucio, CM Gibbs Pickens, C Deneux‐Tharaux, E Ortiz‐Panozo, E Sullivan, E Ota, G Togoobaatar, G Carroli, H Knight, J Zhang, JG Cecatti, JP Vogel, K Jayaratne, MC Leal, M Gissler, N Morisaki, N Lack, OT Oladapo, Ö Tunçalp, P Lumbiganon, R Mori, S Quintana, AD Costa Passos, AC Marcolin, A Zongo, B Blondel, B Hernández, CJ Hogue, C Prunet, C Landman, C Ochir, C Cuesta, C Pileggi‐Castro, D Walker, D Alves, E Abalos, ECD Moises, EM Vieira, G Duarte, G Perdona, I Gurol‐Urganci, K Takahiko, L Moscovici, L Campodonico, L Oliveira‐Ciabati, M Laopaiboon, M Danansuriya, M Nakamura‐Pereira, ML Costa, MR Torloni, MR Kramer, P Borges, PB Olkhanud, R Pérez‐Cuevas, SB Agampodi, S Mittal, S Serruya, V Bataglia, Z Li, M Temmerman, AM Gülmezoglu

**Affiliations:** ^1^ Department of Social MedicineRibeirão Preto Medical SchoolUniversity of São PauloRibeirão PretoSPBrazil; ^2^ Department of Reproductive Health and ResearchUNDP‐UNFPA‐UNICEF‐WHO World Bank Special Programme of ResearchDevelopment and Research Training in Human ReproductionWHOGenevaSwitzerland; ^3^ Research Institute for DevelopmentUniversité Paris DescartesSorbonne Paris Cité, UMR 216ParisFrance; ^4^ Latin American Center for PerinatologyWomen and Reproductive Health, (CLAP/WR)WHO Regional Office for the AmericasMontevideoUruguay; ^5^ Department of EpidemiologyRollins School of Public HealthEmory UniversityAtlantaGAUSA; ^6^ Inserm U1153Obstetrical, Perinatal and Pediatric Epidemiology Research TeamCenter for Epidemiology and BiostatisticsParis Descartes UniversityParisFrance; ^7^ Center for Population Health ResearchNational Institute of Public HealthCuernavacaMexico; ^8^ Faculty of HealthUniversity of TechnologySydneyNSWAustralia; ^9^ Department of Health PolicyNational Center for Child Health and DevelopmentTokyoJapan; ^10^ Centro Rosarino de Estudios PerinatalesRosarioArgentina; ^11^ Royal College of Obstetricians and GynaecologistsOffice for Research and Clinical AuditLindsay Stewart R&D CentreLondonUK; ^12^ Department of Health Services Research and PolicyLondon School of Hygiene and Tropical MedicineLondonUK; ^13^ Ministry of Education–Shanghai Key Laboratory of Children's Environmental HealthXinhua HospitalShanghai Jiao Tong University School of MedicineShanghaiChina; ^14^ Department of Obstetrics and GynaecologySchool of Medical SciencesUniversity of Campinas (UNICAMP)CampinasSPBrazil; ^15^ Family Health BureauMinistry of HealthColomboSri Lanka; ^16^ Escola Nacional de Saúde Pública (ENSP)Fundação Oswaldo Cruz (FIOCRUZ)Rio de JaneiroBrazil; ^17^ National Institute for Health and WelfareHelsinkiFinland; ^18^ Department of PaediatricsGraduate School of MedicineUniversity of TokyoTokyoJapan; ^19^ Bayerische Arbeitsgemeinschaft für Qualitätssicherung in der Stationären Versorgung (BAQ)Bayerische KrankenhausgesellschaftMunichGermany; ^20^ Department of Obstetrics and GynecologyFaculty of MedicineKhon Kaen UniversityKhon KaenThailand; ^21^ Department of Gynaecology and ObstetricsRibeirão Preto Medical SchoolUniversity of São PauloRibeirão PretoSPBrazil; ^22^ Direction de la santé de la familleMinistère de la SantéOuagadougouBurkina Faso; ^23^ Institute for Health Metrics and EvaluationUniversity of WashingtonSeattleWAUSA; ^24^ School of Public HealthHealth Sciences University of MongoliaUlaanbaatarMongolia; ^25^ GLIDE Technical Cooperation and ResearchRibeirão PretoSPBrazil; ^26^ Department of Paediatrics, Ribeirão Preto Medical SchoolUniversity of Sao Paulo, Ribeirão PretoSPBrazil; ^27^ Departments of Obstetrics & Gynaecology and Global Health SciencesUniversity of CaliforniaSan FranciscoCAUSA; ^28^ Department of Biostatistics and DemographyFaculty of Public HealthKhon Kaen UniversityKhon KaenThailand; ^29^ Department of ObstetricsSchool of Medicine of São PauloSão Paulo Federal UniversitySão PauloBrazil; ^30^ Social Protection and Health DivisionInter‐American Development BankMexico CityMexico; ^31^ Fortis Memorial Research InstituteGurgaonHaryanaIndia; ^32^ Hospital Nacional de ItauguáItauguáParaguay

**Keywords:** Benchmarking, caesarean delivery rates, caesarean section rates, logistic regression

## Abstract

**Objective:**

To generate a global reference for caesarean section (CS) rates at health facilities.

**Design:**

Cross‐sectional study.

**Setting:**

Health facilities from 43 countries.

**Population/Sample:**

Thirty eight thousand three hundred and twenty‐four women giving birth from 22 countries for model building and 10 045 875 women giving birth from 43 countries for model testing.

**Methods:**

We hypothesised that mathematical models could determine the relationship between clinical‐obstetric characteristics and CS. These models generated probabilities of CS that could be compared with the observed CS rates. We devised a three‐step approach to generate the global benchmark of CS rates at health facilities: creation of a multi‐country reference population, building mathematical models, and testing these models.

**Main outcome measures:**

Area under the ROC curves, diagnostic odds ratio, expected CS rate, observed CS rate.

**Results:**

According to the different versions of the model, areas under the ROC curves suggested a good discriminatory capacity of C‐Model, with summary estimates ranging from 0.832 to 0.844. The C‐Model was able to generate expected CS rates adjusted for the case‐mix of the obstetric population. We have also prepared an e‐calculator to facilitate use of C‐Model (www.who.int/reproductivehealth/publications/maternal_perinatal_health/c-model/en/).

**Conclusions:**

This article describes the development of a global reference for CS rates. Based on maternal characteristics, this tool was able to generate an individualised expected CS rate for health facilities or groups of health facilities. With C‐Model, obstetric teams, health system managers, health facilities, health insurance companies, and governments can produce a customised reference CS rate for assessing use (and overuse) of CS.

**Tweetable abstract:**

The C‐Model provides a customized benchmark for caesarean section rates in health facilities and systems.

## Tweetable abstract

The C‐Model provides a customized benchmark for caesarean section rates in health facilities and systems.

## Introduction

Caesarean section (CS) is the most commonly performed surgical operation in the world. This surgery is lifesaving when performed in time to overcome certain types of dystocia and other complications. However, as for any major surgery, it presents increased risk of adverse outcomes, including blood transfusion, anaesthesia complications, internal organ injury, infection, thromboembolic disease, neonatal respiratory distress, and other complications of iatrogenic prematurity^1^, ^2^ When carried out without medical indication, there is little benefit added and the harm that can be caused becomes more evident.

Since its introduction in obstetric practice, caesarean section rates have continuously increased in both developed and developing countries.^1^, ^3^, ^4^, ^5^ In 1985, participants of a World Health Organization (WHO) meeting held in Fortaleza, Brazil, stated that CS rates higher than 15% could hardly be justified from a medical standpoint.^6^ Over the years, this figure became the reference for what is considered the ‘ideal’ CS rate. Nevertheless, most countries have observed a steep increase of CS rates in the last three decades.^3^, ^7^, ^8^, ^9^, ^10^, ^11^, ^12^, ^13^ A substantial proportion of this increment was due to unnecessary operations attributable to non‐evidence‐based indications, professional convenience, maternal request, and over‐medicalisation of childbirth^14^. This is an important issue for health systems in many parts of the world, not only because of the additional short‐ and long‐term health risks it causes but also regarding increased costs associated with caesarean births.

Recent data from developed countries suggests that CS rates of around 15% at the population level are possible, safe and compatible with optimum health outcomes for mothers and babies.^15^ However, at the level of an individual health facility, it is often difficult to determine an appropriate rate of CS. Differences in the case‐mix and the obstetric profile complicate the applicability and relevance of a universal reference rate for CS. Based on data disaggregation in ten obstetric groups, Robson proposed in 2001 a classification system that enables understanding of the internal structure of the CS rate at individual health facilities and identification of strategic population groups to prevent unnecessary use of CS.^16^, ^17^, ^18^ In 2015, the WHO issued an official statement concerning CS rates and promoting the use of the Robson classification as an tool for optimising the CS rate at health facilities.^19^

Building on the clinical‐obstetric characteristics that form the base of the Robson classification, we carried out this study with the objective of developing and testing a global reference for CS rates at health facilities.

## Methods

**Figure 1:**
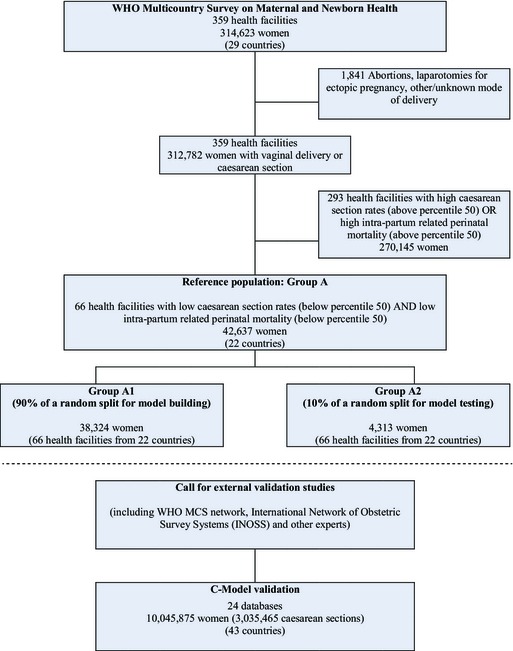
Analysis flowchart for model building and validation.

We hypothesised that mathematical models could determine the relationship between clinical‐obstetric characteristics and CS. These models would be able to generate probabilities of CS that could be compared with observed CS rates. This approach is widely accepted for benchmarking performance of intensive care units. In intensive care, mathematical models are used to estimate the probability of mortality and this information is compared with the actual mortality.^20^ Thus, we devised a three‐step approach to generate the global benchmark of CS rates at health facilities: (1) creation of a multi‐country reference population; (2) building mathematical models; (3) testing mathematical models with available multicentre facility‐based data in various countries, contexts and health systems. The overall analysis flow is presented in Figure 1.

### Building a reference population

A critical step for the development of a mathematical model able to generate a reference CS rate is the population that will serve as the basis for establishing the relationship between maternal characteristics and the probability of caesarean section. Due to the global nature of our endeavour, we assumed we would need a multi‐country population, with relatively low CS rates and, at the same time, good outcomes of labour and childbirth. We used the WHO Multi‐Country Survey on Maternal and Newborn Health (WHO MCS) to create our reference population. Detailed description of this study has been published elsewhere.^21^ Briefly, the WHO MCS was a cross‐sectional study implemented in 359 health facilities in 29 countries. Countries, provinces (or other equivalent political divisions within countries), and health facilities were randomly selected to participate in the WHO MCS through a stratified, multistage cluster sampling strategy. Health facilities were only eligible if they dealt with at least 1000 deliveries per year and had the capacity to provide CS. Between May 2010 and December 2011 the WHO MCS included 314 623 women from Africa, Asia, the Eastern Mediterranean region, and Latin America.

For the creation of the reference population we considered that the intrapartum related perinatal mortality (i.e. intrapartum stillbirth plus neonatal deaths that took place in the first postpartum day) was a reasonable indicator of quality of care.^22^ We assumed that health facilities with low CS rates and low intrapartum perinatal mortality had few unnecessary CS and good maternal and perinatal outcomes. Therefore, we selected this population to serve as reference and base for mathematical modelling. To implement this reasoning, we first calculated the facility‐specific CS rate and intrapartum‐related perinatal mortality rate in each of the 359 health facilities that participated in the WHO MCS. Next, we identified the health facilities that would have both low CS rates and low intrapartum perinatal mortality. Low CS rates and low intrapartum perinatal mortality were both relative to the facilities that participate in the WHO MCS and defined as below the respective percentile 50. We selected this specific cut‐off (i.e. percentile 50) because the median is commonly used as a reference for defining what is low or high in sufficiently large samples. Thus, hospitals that presented both CS rates and intrapartum‐related perinatal mortality below the 50th percentile constituted the reference population (in Supporting Information Appendix S1, Figure S2, Group ‘A’). We used simple frequencies and proportions to describe essential characteristics of the reference population.

### Building the C‐Model

The WHO MCS collected data on various socio‐demographic and clinical‐obstetric characteristics, severity markers, and complications that were analysed as candidate predictors for CS. First, we assessed the reference population and the WHO MCS country data using the Robson classification. Secondly, we carried out univariate analyses of the reference population to explore the relationship of several candidate predictors and the occurrence of CS. Third, a logistic regression random effects model was used to determine the relationship between candidate predictors and CS. As part of the regression analysis, the reference population was divided in two subpopulations: ‘A1’ (used for model building), and ‘A2’ (used for testing the internal validity of the prediction model).^23^ To maximise representativeness and at the same time ensure the ability to test the internal validity of the model, 90% of the reference population was randomly allocated to population ‘A1’ and the remainder (10%) to population ‘A2’.

We named our mathematical model ‘C‐Model’ and built four versions to be used according to the availability of data at the local level. The first version has a limited number of variables and is essentially based on the variables used in the Robson classification (i.e. parity, previous CS, multiple pregnancy, provider‐initiated childbirth, presentation, and preterm birth). The second version added a demographic variable (i.e. maternal age), the third one the presence of organ dysfunction or ICU admission, and the fourth variables of diagnosis of selected complications (i.e. placenta praevia, abruptio placenta, chronic hypertension, pre‐eclampsia, renal disease and HIV). Only variables that contributed significantly to each model version were retained. The different versions of C‐Model estimate the probability of CS based on the presence or absence of significant predictors.^23^

### Testing the C‐Model

The internal validity of C‐Model was assessed in subpopulations A1 and A2. The discriminatory power was assessed through ROC curves. Classification tables, accuracy tests, and model performance tests were also carried out.

An international call for validation studies of the C‐Model was issued in February 2014. Members of the WHO MCS research network, the International Network of Obstetric Surveys Systems (INOSS), and independent researchers received an invitation to test the C‐Model in their databases. To ensure standardisation, an external validation protocol with detailed instructions including variable recoding, computing probabilities of CS using the C‐Model coefficients, generating ROC curves, and other information were provided. The researchers that responded to the call returned their results via e‐mail and the information was compiled in a master spreadsheet (Supporting Information Appendix S2). For each database, estimates generated by C‐Model versions with incomplete data (i.e. when a variable was not available in the dataset) were discarded. Random effects meta‐analyses were used to generate summary estimates of areas under the ROC curves. Based on the results of these meta‐analyses, the C‐Model version with largest number of variables was considered able to generate the ‘best estimate’ of CS probability. The deviation of the observed CS rate (and an uncertainty range) was determined for each database considering the best estimate of CS probability. This uncertainty range was arbitrarily defined by the authors as 20% because differences >20–25% are commonly considered clinically significant or appreciable differences.^24^ The ratio between observed and predicted CS (the standardised caesarean section ratio) was determined.

All analysis considered cluster effect at the health facility level and at the country level. STATA Version 13.0 (www.stata.com/stata13/), PASW Statistics 18, Release Version 18.0.0 (SPSS, Inc., 2009, Chicago, IL, USA, www.spss.com), R package version 3.1.1 (http://www.r-project.org/) and MEDCALC Version 11.6.1.0 (MedCalc Software, 2011, Mariakerke, Belgium, www.medcalc.org) were used in the C‐Model analysis.

The HRP specialist panel (WHO scientific staff and external, independent researchers) on epidemiological research reviewed and approved the WHO MCS study protocol for technical content. The WHO MCS study was approved by the WHO ethical review committee and the relevant ethical clearance mechanisms in all countries. The relevant ethical and administrative clearances for analysis of databases that provided data for C‐Model building and testing were obtained.

## Results

A total of 359 health facilities in 29 countries participated in the WHO MCS, which included 314 623 women between 1 May 2010, and 31 December 2011. Among these health facilities, the 50th percentile for CS rate was 30% and the 50th percentile for the intrapartum‐related perinatal deaths was 6.8 deaths per 1000 livebirths. Health facilities below these values (i.e. facilities with <30% of caesarean births and <6.8 intrapartum‐related perinatal deaths deaths per 1000 births) were selected to provide the reference population (Group A). The reference population included 42 637 women from 66 health facilities in 22 countries. Table 1 presents the demographic characteristics of health indicators and countries of the reference population. Table 2 presents the results of the Robson classification in the reference population. Supporting Information Table S1 presents the results of univariate analysis of the relationship of CS and selected characteristics in the Group A; these characteristics were later included in different versions of the C‐Model.

**Table 1:** Characteristics and outcomes of the reference population (Group A), including health facilities and countries^a^

	All women (*n *= 42 637)
**Age**	
Data available	42 551
Mean (SD)	27.0 years (±5.9)
**Marital status**	
Data available	41 022
Without a partner	4973 (12.1%)
With a partner	36 049 (87.9%)
**Schooling**	
Data available	36 770
Mean (SD)	9.7 years (±4.4)
**Outcomes**	
Livebirths	42 361
Caesarean sections	7629 (17.9%)
Perinatal deaths	276 (6.5/1000 livebirths)
Intrapartum‐related perinatal deaths	127 (3.0/1000 livebirths)
Maternal deaths	10 (23.6/100 000 livebirths)
Maternal near‐miss cases	111 (2.6/1000 livebirths
Severe maternal outcomes	121 (2.9/1000 livebirths)
Case‐fatality ratio	11:1
**Health facilities**	
Data available	62 health facilities
Location (%)	
Urban	31 (50.0)
Peri‐urban	15 (24.2)
Rural	16 (25.8)
Level of care (%)	
Primary	13 (21.0)
Secondary	33 (53.2)
Tertiary	15 (24.2)
Other	1 (1.6)
Teaching hospital (%)	
Yes	38 (61.3)
No	24 (38.7)
Maternity beds (%)	
<50	46 (74.2)
50–100	10 (16.3)
>100	6 (9.7)
**Countries** (%)	
Data available	42 637
Afghanistan	2102 (4.9)
Angola	1808 (4.2)
Argentina	2507 (5.9)
Brazil	1555 (3.6)
Cambodia	1980 (4.6)
China	1053 (2.5)
Ecuador	216 (0.5)
India	2007 (4.7)
Japan	3096 (7.3)
Mongolia	5101 (12.0)
Nepal	490 (1.1)
Nicaragua	41 (0.1)
Nigeria	924 (2.2)
Palestine	980 (2.3)
Pakistan	497 (1.2)
Peru	637 (1.5)
Philippines	4391 (10.3)
Qatar	3950 (9.3)
Sri Lanka	3973 (9.3)
Thailand	842 (2.0)
Uganda	1791 (4.2)
Vietnam	2696 (6.3)

a Considering the 359 health facilities that participated in the WHO MCS, the reference group is composed of those with caesarean section rates and perinatal mortality below the 50th percentile): 66 health facilities with low caesarean section rates and low perinatal mortality from 22 countries.

**Table 2:** Description of the reference population (Group A) according to Robson's classification

Group	Description	CS/group	C‐Section rate, %	Relative size, %	CS/all births, %
1	Nulliparous women with a single cephalic pregnancy, at greater than or equal to 37 weeks in spontaneous labour	1182/12 069	9.8	29.3	2.9
2	Nulliparous women with a single cephalic pregnancy, at greater than or equal to 37 weeks’ gestation who either had labour induced or were delivered by caesarean section before labour (provider‐initiated childbirth)	1446/3620	39.9	8.8	3.5
3	Multiparous women, without a previous uterine scar, with a single cephalic pregnancy at greater than or equal 37 weeks in spontaneous labour	503/16 538	3.0	40.1	1.2
4	Multiparous women, without a previous uterine scar, with a single cephalic pregnancy at greater than or equal to 37 weeks’ gestation who either had labour induced or were delivered by caesarean section before labour (provider‐initiated childbirth)	624/2631	23.7	6.4	1.5
5	All multiparous women, with at least one previous uterine scar and a single cephalic pregnancy at greater than or equal to 37 weeks’ gestation	2194/2948	74.4	7.2	5.3
6	All nulliparous women with a single breech pregnancy	391/498	78.5	1.2	0.9
7	All multiparous women with a single breech pregnancy including women with previous uterine scars	471/638	73.8	1.5	1.1
8	All women with multiple pregnancies, including women with previous uterine scars	222/385	57.7	0.9	0.5
9	All women with a single pregnancy with a transverse or oblique lie, including women with previous uterine scars	140/158	88.6	0.4	0.3
10	All women with a single cephalic pregnancy at less than or equal to 36 weeks’ gestation, including women with previous scars	432/1718	25.1	4.2	1.0
Overall		7605/41 203	18.5	100.0	18.5

Number of missing values = 1434.

Random split of the reference population rendered two subgroups: sub‐group A1 with 38 324 women and subgroup A2 with 4313 women. The mixed‐effects logistic regression modelling was carried out in subgroup A1 and produced four versions of the C‐Model, all of them computing the clustering effect. Table 3 presents the variables included in each model together with their coefficients and other relevant details. These versions were tested in subgroup A2. Overall, in subgroup A1, areas under ROC curves ranged from 0.867 to 0.879, and in subgroup A2 from 0.873 to 0.886. The diagnostic odds ratio of the C‐Model (determined based on cut‐off points derived from ROC curves) ranged from 17.49 to 18.54 in subgroup A1 and from 18.12 to 19.11 in subgroup A2. The percentage of cases correctly classified ranged from 82.2 to 83.6% in subgroup A2, according to the different version of the model. Visual assessment of calibration plots (based on quintiles of C‐Model caesarean‐section probabilities) in both subgroups indicates satisfactory performance, with predicted CS rates following observed CS rates. Appendix S1 contains supplementary information related to the model building and testing, including ROC curves, diagnostic accuracy, classification tables, calibration plots and other tests whose results suggested satisfactory model performance (for details refer to Supporting Information Figures S3–S5 and Tables S5–S8).

**Table 3:** C‐Model coefficients (four versions depending on the availability of data)

			v1.0	v1.1	v1.2	v1.3
**Constant**		(*β*)	–3.392134	–3.992549	–3.989357	–4.015252
**Covariates**						
Obstetric characteristics						
(*x*_1_)	Parity	(*β*_1_)	–0.559968	–0.760441	–0.76173	–0.77531
(*x*_2_)	Previous C‐section	(*β*_2_)	2.842534	2.873179	2.87813	2.922222
(*x*_3_)	Multiple pregnancy	(*β*_3_)	1.694844	1.722743	1.721366	1.834027
(*x*_4_)	Provider‐initiated childbirth^a^	(*β*_4_)	2.747953	2.708164	2.686502	2.634921
(*x*_5_)	Presentation	(*β*_5_)	2.922391	2.911992	2.9241	2.985162
(*x*_6_)	Preterm birth	(*β*_6_)	0.368073	0.364223	0.285275	–
Demographics and severity						
(*x*_7_)	Maternal age	(*β*_7_)	–	0.734265	0.728236	0.71104
(*x*_8_)	Organ dysfunction OR ICU admission	(*β*_8_)	–	–	1.499462	0.661417
Complications						
(*x*_9_)	Placenta praevia	(*β*_9_)	–	–	–	3.796513
(*x*_10_)	Abruptio placentae	(*β*_10_)	–	–	–	2.741255
(*x*_11_)	Chronic hypertension	(*β*_11_)	–	–	–	0.561991
(*x*_12_)	Pre‐eclampsia	(*β*_12_)	–	–	–	0.98718
(*x*_13_)	Renal disease	(*β*_13_)	–	–	–	1.301346
(*x*_14_)	HIV	(*β*_14_)	–	–	–	1.310211
**Determining the probability of caesarean section** b						
Rules: In general, if condition is absent, make x_i_ = 0; if condition is present, make x_i_ = 1; For presentation, if cephalic, make *x*_5_ = 0; if breech, make *x*_5_ = 1; if transverse lie or other presentation, make *x*_5_ = 2; For pre‐eclampsia, if absent, make *x*_12_ = 0; in case of pre‐eclampsia, make *x*_12_ = 1; in case of eclampsia, make *x*_12_ = 2 Calculate Logit, using the relevant coefficients for each model, as follows: Logit = *β* + (*x*_1_ *β*_1_) + (*x*_2_ *β*_2_) + … + (*x*_*i*_ *β*_*i*_) Calculate the probability of caesarean section ProbCS = e^Logit^/(1 + e^Logit^)						

a Includes both induction of labour and caesarean section before labour.

b An electronic calculator is through the link www.who.int/reproductivehealth/publications/maternal_perinatal_health/c-model/en/

The C‐Model was tested in 24 independent databases (Supporting Information Tables S3–S9) including data of over 10 million women and 3 million CS performed in 43 countries. Figure S1 and Table S4 represent all countries where the C‐Model was tested. According to the different versions of the model, areas under the ROC curves suggested a good discriminatory capacity of C‐Model, with summary estimates ranging from 0.832 to 0.844 (Table S2). Appendix S1 presents areas under the ROC curves for individual databases and meta‐analysis details. Table S3 presents the CS rate, the best estimate of predicted CS rate (with a reference range of CS rate), the absolute deviation of the best estimate, and the standardised CS ratio by database that tested the C‐Model. The C‐Model was able to generate expected CS rates that are adjusted for the case‐mix of the obstetric population. In databases with low CS rates and good perinatal outcomes (e.g. Finland) the deviation from observed and estimated CS rates is minimal (i.e. <1.0%). Hospitals with high CS rates presented larger deviations from what would be expected for the clinical and obstetric characteristics of the population. Detailed results of the studies that conducted external validation analysis are presented in the Appendix S1 (Figures S6–S9) and Appendix S2.

## Discussion

### Main findings

This article describes the development of a global reference for benchmarking CS rates at health facilities. Based on maternal characteristics, the C‐Model is able to generate an individualised reference rate for CS for health facilities and groups of health facilities, as well as the probability of a woman of having a CS in a particular facility. This tool was developed based on a multicountry population and tested with data from over 10 million women from 43 countries. With the C‐Model, obstetric teams, health facilities, health system managers, health insurance organisations, and governments will be able to obtain a customised reference rate for CS; this reference rate is adapted to the clinical, demographical, and obstetric profile of the maternal population. This tool provides an objective estimate to assess, compare, and drive C‐section rates, locally and nationally. To facilitate use, an electronic calculator was developed and made available through the link www.who.int/reproductivehealth/publications/maternal_perinatal_health/c-model/en/. This online calculator not only provides estimates of CS probabilities for individual women, but also generates estimates for facility databases that can be uploaded in various formats into the free online system (see Appendix S1).

### Strengths and limitations

This is the largest study aiming at establishing the mathematical relationship of maternal characteristics and CS, with a large sample size, multicountry data and extensive external testing. Another positive feature of our approach is that, depending on local data availability, more sophisticated versions of the model can be used. It should be noted that the gain in accuracy between the different versions of the model is small, possibly because the most basic version of the model already includes the main predictors of C‐section.

Some weaknesses of this analysis should be noted. First, we generated the reference population using a low risk sub‐sample of the WHO MCS database. Despite efforts to ensure the best possible quality in this database (considering the scope and magnitude of the original study) minor data heterogeneity could be expected and has been discussed in previous publications^21^. For the same reason, we were not able to include in our models all possible predictors of CS. Some important information, such as maternal weight and height, were not available in the WHO MCS database and thus were not considered. At this point it is uncertain whether the included predictors have compensated (at least partially) for the absence of other important predictors and how these other predictors compare with the included ones.

Secondly, as we were intending to use individual maternal characteristics to estimate C‐section probabilities, we did not include in our modelling the characteristics of health facilities, which could represent a limitation for generating estimates in health facilities with severe shortages of essential supplies, human resources or other factors that are necessary to perform safe CS. Similarly, the C‐Model estimates do not account for CS performed for convenience (e.g. maternal request, health professional or health service preference), so that mostly medically necessary CS are modelled and counted. In any case, when the C‐Model estimates show appreciable differences from actual CS rates, this could trigger further inquiries and actions to improve quality of care, decision‐making, practices, and results.

Thirdly, most of the validation data comes from routine databases, which may add some issues concerning completeness and accuracy of the information on clinical risk factors in those databases. In addition, these routine data could be associated to some heterogeneity as some health facilities or countries may not respond uniformly to maternal characteristics and CS predictors; we tried to account for this heterogeneity by suggesting the use of ranges of uncertainty for each estimate.

Finally, ‘Induction of labour’ and ‘C‐section before labour’ were treated as ‘provider‐initiated childbirth’. This was performed in line with the Robson classification that lumps together in the same category induction of labour and CS before labour. We assumed that as a reference to all childbirths, the C‐Model should be able to generate estimates to all obstetric population, including those women with CS before labour. As we could not generate a coefficient specific to CS before labour (it would have a 100% association with CS), we decided to consider induction of labour as a reasonable proxy for CS before labour during model development. This decision partly accounts for the provider intent of ending the pregnancy while also considering that many women with an indication to end the pregnancy might actually undergo induction of labour first instead of undergoing CS before labour.

### Interpretation

This analysis builds on previous efforts to establish the relationship of maternal characteristics and CS. These efforts have explored, among others, comparisons of caesarean rates across different populations and institutions, application of dynamic econometric models to assess aggregate level determinants of caesarean section rates in developed countries, adjustments for Robson's Ten‐Group Classification System (TGCS), and clinical and socio‐demographic variables of the mother and the fetus for inter‐hospital comparisons of CS rates.^10^, ^13^, ^25^, ^26^ However, whereas previous approaches tended to be limited in terms of the number of countries and global representativeness, the present work used a multicountry reference population and extended testing to maximise global applicability. Based on our findings, we believe we have developed a robust and potentially useful tool to improve the ability of health facilities and systems to objectively estimate the overuse and under‐use of caesarean sections and use this information to motivate change.

## Conclusion

Potential applications of the C‐Model include its use as part of clinical audits and negotiated targets within health services. Countries could set their reference CS rate based on the obstetric profile of their population and not depend on a single ideal CS rate. However, caution should be exercised: C‐Model estimates should not be used to guide decision making in individual clinical practice and should not replace clinical judgment (e.g. an individual woman may need a CS despite a C‐Model estimate suggesting very low probability of CS). Researchers around the world are encouraged to further test the C‐Model and report their findings, particularly as part of strategies designed to optimise the use of CS in health services. It would be desirable to develop alternative models and, if possible, test other potential predictors such as maternal height, maternal weight, and BMI. Studies on equitable use of CS could also use the C‐Model to estimate over‐use and under‐use of CS in specific populations, such as low‐income or low‐education populations.

Although there is a global trend towards increased rates of CS, under‐use of this intervention remains an issue in many countries, particularly among underprivileged populations. The C‐Model is a tool designed to guide obstetric teams, health managers, and other stakeholders in the complex task of optimising the use of CS. Through a customised estimate of CS rates, the C‐Model may provide a locally relevant reference of what would be an optimal CS rate.

### Declaration of interests

None declared.

### Contribution to authorship

This project is the result of an international collaborative effort by a large group of institutional and researcher members of the C‐Model Development Group. JPS conceptualised the analysis plan and drafted the report on behalf of the C‐Model Development group.

JPS, APB, AD, BM, CMGP, CD‐T, EOP, EO, GT, GC, HK, JZ, JGC, JPV, KJ, MCL, MG, NM, NL, OTO, OT, PL, RM, SQ, AMG: Made substantial contributions to the conception or design of the work, or the acquisition, analysis, or interpretation of data for the work.

All authors revised the work critically; gave final approval of the version to be published; agreed to be accountable for all aspects of the work in ensuring that questions related to the accuracy or integrity of any part of the work are appropriately investigated and resolved.

LO‐C led the development of the electronic calculator. All members of the C‐Model Development Group read and approved the final manuscript.

### Details of ethics approval

The HRP specialist panel (WHO scientific staff and external, independent researchers) on epidemiological research reviewed and approved the WHO MCS study protocol for technical content. The WHO MCS study was approved by the WHO ethical review committee and the relevant ethical clearance mechanisms in all countries. The relevant ethical and administrative clearances for analysis of databases that provided data for C‐Model building and testing were obtained.

### Funding

This project was supported through the in‐kind collaboration of the many institutions that host the members of the C‐Model development group. There was no specific financial support for this study.

## Supporting information


**Appendix S1.**
**Figure S1.** The 43 countries that provided data for the C‐Model building and testing.
**Figure S2.** Stratification of health facilities based on the percentile 50 of caesarean section rates (30%) and intrapartum related perinatal deaths (6 deaths/1000 live births).
**Figure S3.** ROC Curves for models designed to estimate the probability of Caesarean section.
**Figure S4.** Forest plot of areas under the ROC curves (model building and testing).
**Figure S5.** Predicted (―blue line) and observed (―red line) caesarean section rates by population quintiles of Caesarean section probabilities, in the Groups A1 and A2.
**Figure S6.** Meta‐analysis of external validation studies for the model v1.0 with 95% confidence intervals (i2 = 99·96%, studies ordered by the size of study population).
**Figure S7.** Meta‐analysis of external validation studies for the model v1.1 with 95% confidence intervals (i2 = 99·96%, studies ordered by the size of study population).
**Figure S8.** Meta‐analysis of external validation studies for the model v1.2 with 95% confidence intervals (i2 = 99·92%, studies ordered by the size of study population).
**Table S1.** Univariate analysis of Caesarean section predictors included in the C‐Model (Group A)^a,b^.
**Table S2.** The external validation of C‐Model (summary estimates of areas under the ROC curves with 95% confidence intervals; random effects meta‐analyses).
**Table S3.** C‐Model calibration and use as benchmark for caesarean section rates (ordered by crescent deviation of the best estimate of average caesarean section probability).
**Table S4.** The 43 countries that provided data used to develop and test the C‐Model.
**Table S5.** Classification table for assessment of the diagnostic accuracy (with 95% confidence intervals) and the percentage of cases correctly classified by models designed to estimate the probability of caesarean section*^§^.
**Table S6.** Indicators of model performance.
**Table S7.** C‐Model coefficients estimated using the R package*.
**Table S8.** Sensitivity analysis including only databases with complete data for all C‐Model versions. (summary estimates of areas under the ROC curves with 95% confidence intervals; random effects meta‐analyses).
**Table S9.** Sensitivity.

**Appendix S2.** Spreadsheet with compilation of results of external validation studies.
