# Supplementary material for: A global reference for caesarean section rates (C‐Model): a multicountry cross‐sectional study
Source: BJOG. 2015 Aug 10;123(3):427–36. doi: 10.1111/1471-0528.13509 (PMC4873961; doi:10.1111/1471-0528.13509)
Supplement: Supplementary file 1 — Appendix S1. Figure S1. The 43 countries that provided data for the C‐Model building and testing. Figure S2. Stratification of health facilities based on the percentile 50 of caesarean section rates (30%) and intrapartum related perinatal deaths (6 deaths/1000 live births). Figure S3. ROC Curves for models designed to estimate the probability of Caesarean section. Figure S4. Forest plot of areas under the ROC curves (model building and testing). Figure S5. Predicted (―blue line) and observed (―red line) caesarean section rates by population quintiles of Caesarean section probabilities, in the Groups A1 and A2. Figure S6. Meta‐analysis of external validation studies for the model v1.0 with 95% confidence intervals (i2 = 99·96%, studies ordered by the size of study population). Figure S7. Meta‐analysis of external validation studies for the model v1.1 with 95% confidence intervals (i2 = 99·96%, studies ordered by the size of study population). Figure S8. Meta‐analysis of external validation studies for the model v1.2 with 95% confidence intervals (i2 = 99·92%, studies ordered by the size of study population). Table S1. Univariate analysis of Caesarean section predictors included in the C‐Model (Group A)a,b. Table S2. The external validation of C‐Model (summary estimates of areas under the ROC curves with 95% confidence intervals; random effects meta‐analyses). Table S3. C‐Model calibration and use as benchmark for caesarean section rates (ordered by crescent deviation of the best estimate of average caesarean section probability). Table S4. The 43 countries that provided data used to develop and test the C‐Model. Table S5. Classification table for assessment of the diagnostic accuracy (with 95% confidence intervals) and the percentage of cases correctly classified by models designed to estimate the probability of caesarean section*§. Table S6. Indicators of model performance. Table S7. C‐Model coefficients estimated using the R package*. Table S8. Sensitivity analysis [file BJO-123-427-s001.pdf]

Appendix S1.

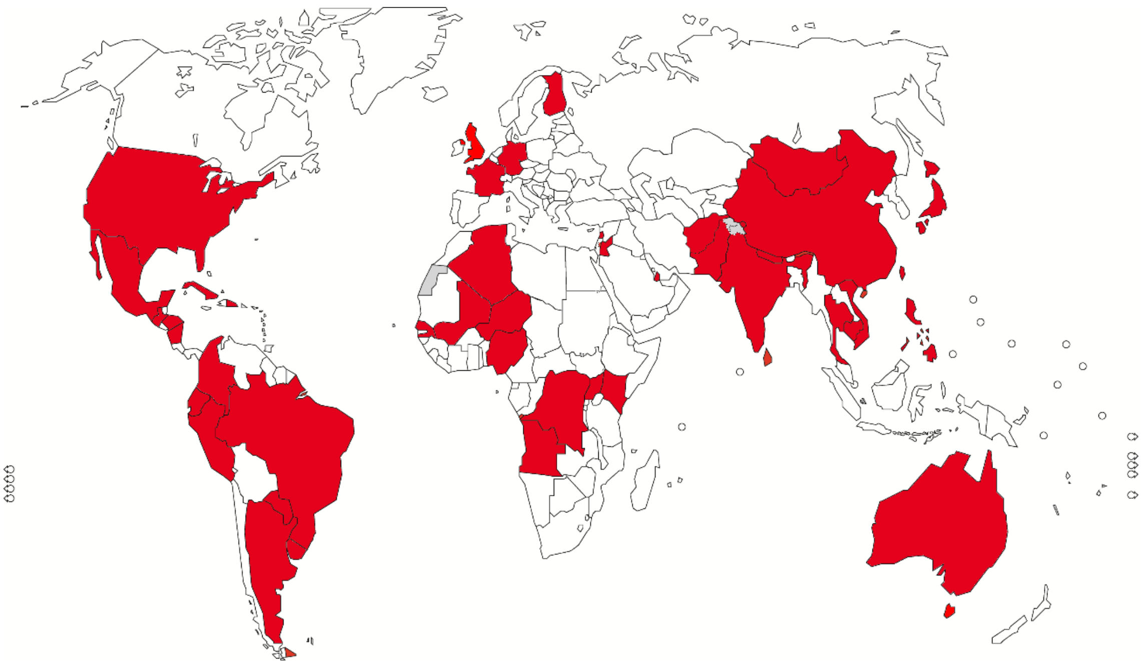

Figure S1. The 43 countries that provided data for the C-Model building and testing

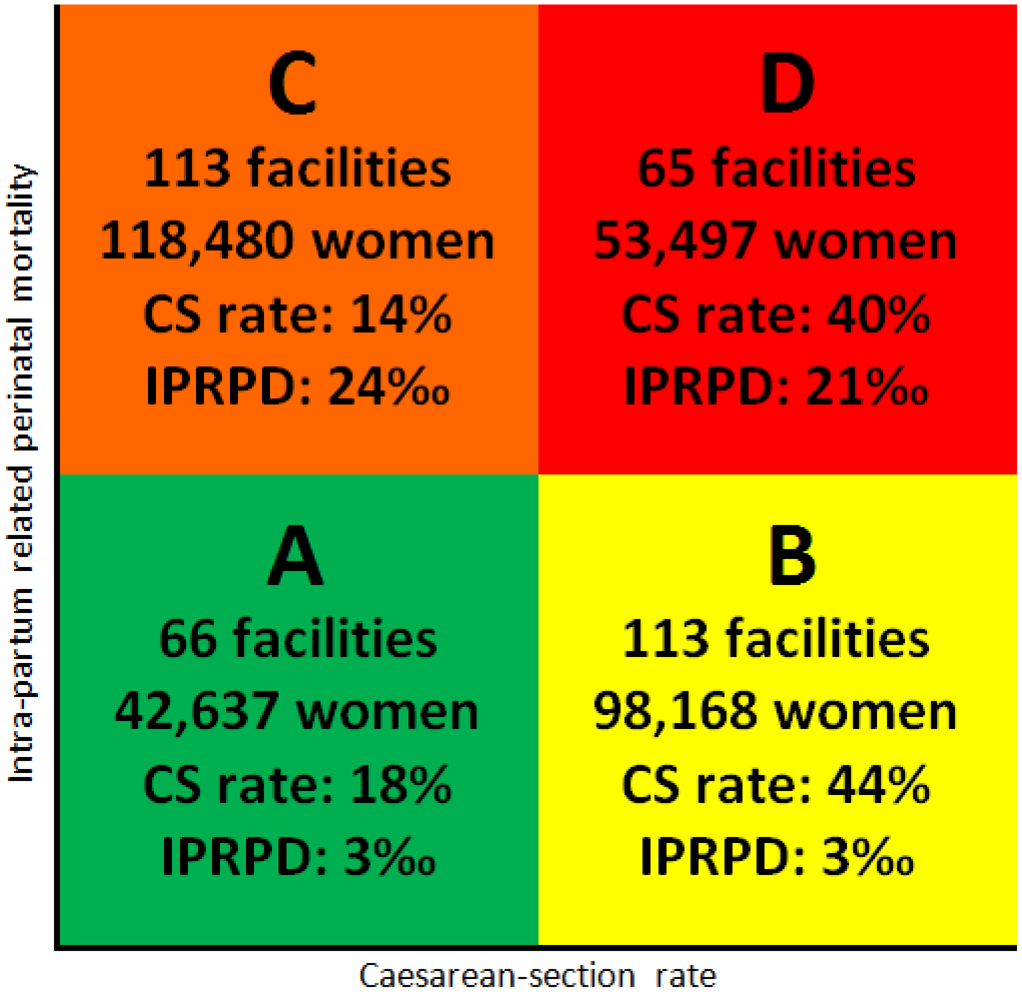

Figure S2. Stratification of health facilities based on the percentile 50 of caesarean section rates (30%) and intrapartum related perinatal deaths (6 deaths /1000 live births). Average values presented for the populations A, B, C and D.

Table S1. Univariate analysis of Caesarean section predictors included in the C-Model (Group A)[a,b]

		All women (N=42,637)	Women with vaginal delivery (n=35,008)	Women with Caesarean section (n=7,629)	p-value (adjusted for cluster effect)
**Obstetric Characteristics**					
Parity	0	17,527	14,188 (80·9%)	3339 (19·1%)	
	1-2	18,701	15,222 (81·4%)	3479 (18·6%)	0·0419
	3+	6,323	5,518 (87·3%)	805 (12·7%)	
Previous C-section	0	37,546	32,721 (87·1%)	4825 (12·9%)	
	1	2,783	824 (29·6%)	1959 (70·4%)	<0·0001
	2+	880	50 (5·7%)	830 (94·3%)	
Multiple pregnancy	–	42,247	34,840 (82·5%)	7407 (17·5%)	<0·0001
	+	385	163 (42·3%)	222 (57·7%)	
Provider-initiated childbirth*	–	33,249	30,459 (91·6%)	2790 (8·4%)	<0·0001
	+	9,364	4,530 (48·4%)	4834 (51·6%)	
Abnormal Presentation	–	41,241	34,693 (84·1%)	6548 (15·9%)	<0·0001
	+	1,209	284 (23·5%)	925 (76·5%)	
	#	164	18 (11%)	146 (89%)	
Preterm birth	–	39,318	32,327 (82·2%)	6991 (17·8%)	<0·0001
	+	2,034	1,406 (69·1%)	628 (30·9%)	
**Demographics and Severity**					
Maternal Age	<20	3,803	3,423 (90%)	380 (10%)	
	20-34	33,348	27,666 (83%)	5682 (17%)	<0·0001
	>35	5,400	3,845 (71·2%)	1555 (28·8%)	
Organ Dysfunction or ICU	–	42,386	34,930 (82·4%)	7456 (17·6%)	<0·0001
	+	251	78 (31·1%)	173 (68·9%)	
**Complications**					
Placenta Praevia	–	42,513	34,993 (82·3%)	7520 (17·7%)	<0·0001
	+	124	15 (12·1%)	109 (87·9%)	
Abruptio Placentae	–	42,527	34,984 (82·3%)	7543 (17·7%)	<0·0001
	+	110	24 (21·8%)	86 (78·2%)	
Chronic hypertension	–	42,451	34,916 (82·3%)	7535 (17·7%)	<0·0001
	+	186	92 (49·5%)	94 (50·5%)	
Preeclampsia	–	41,809	34,632 (82·8%)	7177 (17·2%)	
	+	784	362 (46·2%)	422 (53·8%)	<0·0001
	£	44	14 (31·8%)	30 (68·2%)	
Renal disease	–	42,603	34,996 (82·1%)	7607 (17·9%)	<0·0001
	+	34	12 (35·3%)	22 (64·7%)	
HIV	–	42,472	34,857 (82·1%)	7615 (17·9%)	<0·2904
	+	165	151 (91·5%)	14 (8·5%)	

[a] Considering the 359 health facilities that participated in the WHO MCS, the reference group is composed of those with caesarean section rates and perinatal mortality below the percentile 50): 66 health facilities with low caesarean section rates and low perinatal mortality from 22 countries

[b] Missing data (n;%): Parity (86;0·2%), Previous C-section (1428;3·3%), Multiples (5;0·0%), Induction of labour (24;0·1%), Presentation (23;0·1%), Preterm birth (1285;3·0%), Maternal age (86;0·2%), Organ dysfunction or ICU admission (0;0%), Placenta praevia (0;0%), abruption placentae (0;0%), chronic hypertension (0;0%), preeclampsia (0;0%), renal disease (0;0%), HIV (0;0%)

- : absent

+: present

#: transverse lie/other

£: eclampsia

*: includes both induction of labour and caesarean section before labour

Table S2: The external validation of C-Model (summary estimates of areas under the ROC curves with 95% confidence intervals; random effects meta-analyses).

	**AUC**	**Lower bound**	**Upper bound**
**All data**			
C-Model v1.0	0·832	0·806	0·857
C-Model v1.1	0·837	0·814	0·861
C-Model v1.2	0·842	0·823	0·862
C-Model v1.3	0·844	0·826	0·863

Table S3. C-Model calibration and use as benchmark for caesarean section rates (ordered by crescent deviation of the best estimate of average caesarean section probability)

Database (N)	Caesarean section rate (Observed Caesarean sections)	Best Estimate of Predicted Caesarean Section Rate (Predicted Caesarean sections)	Reference range of Caesarean Section rate	Absolute Deviation of Best Estimate	Standardized Caesarean Section Ratio
**Finland**	16·3%	16·0%	13% - 19%	0·3%	1·02
(413871)	(67557)	(66261)			
**Sri Lanka**	26·8%	27·1%	22% - 32%	0·3%	0·99
(496)	(133)	(135)			
**France**	20·2%	19·8%	16% - 24%	0·4%	1·02
(14681)	(2965)	(2901)			
**Argentina**	33·6%	35·3%	28%-48%	1·8%	0·95
(6111)	(2052)	(2160)			
**Brazil - SAMM**	48·1%	50·7%	41% – 61%	2·6%	0·95
(9555)	(4597)	(4845)			
**WHO GS Africa**	12·1%	8·1%	7% - 10%	4·0%	1·49
(83079)	(10067)	(6755)			
**England**	23·7%	18·3%	15% - 22%	5·4%	1·29
(562375)	(133315)	(103140)			
**Australia**	31·8%	26·1%	21% - 31%	5.6 %	1·22
(1472085)	(467969)	(384804)			
**USA-2010 Natality File**	31·9%	25·4%	20% - 31%	6·4%	1·25
(2892041)	(927758)	(740374)			
**USA-2012 Natality File**	32·0%	25·4%	20% - 30%	6·4%	1·26
(3283819)	(1058853)	(838211)			
**Brazil-Mater**	26·7%	20·4%	16% – 24%	6·4%	1·31
(13164)	(3520)	(2683)			
**USA - Consortium on Safe Labour**	29·4%	22·9%	18% - 27%	6·6%	1·29
(206969)	(60866)	(47293)			
**Vietnam**	28·5%	20·0%	16% - 24%	8·5%	1·42
(3056)	(871)	(612)			
**Mali & Senegal**	21·1%	11·5%	9% - 14%	9·6%	1·83
(390155)	(82233)	(44907)			
**WHO GS Asia**	27·5%	15·4%	12% - 18%	12·1%	1·78
(109101)	(29963)	(16813)			
**WHO MCS**	30·3%	17·1%	14% - 21%	13·2%	1·77
(270145)	(81880)	(46195)			
**Mexico**	23·7%	9·9%	8% - 12%	13·9%	2·40
(1310)	(311)	(130)			
**Mongolia**	28·1%	13·1%	10% - 16%	15·0%	2·15
(29215)	(8223)	3831			
**CLAP**	35·8%	19·9%	16% - 24%	15·9%	1·80

(3593)	(1285)	(714)			
**Germany**	34·6%	17·5%	14% - 21%	17·1%	1·98
(104051)	(35983)	(18209)			
**Brazil–CAISM**	46·6%	29·0%	23% – 35%	17·6%	1·61
(12771)	(5957)	(3709)			
**WHO GS LA**	35·6%	17·5%	14% - 21%	18·1%	2·04
(98011)	(34913)	(17143)			
**Brazil ("Birth in Brazil" project)**	56·3%	27·7%	22 – 33%	28·6%	2.03
(23366)	(13166)	(6472)			
**Thailand**	52·3%	20·1%	16% - 24%	32·3%	2·61
(300)	(157)	(61)			

Table S4. The 43 countries that provided data used to develop and test the C-Model

Afghanistan	France	Niger
Angola	Germany	Nigeria
Argelia	Guatemala	Occupied Palestinian Territory
Argentina	Honduras	Pakistan
Australia	India	Paraguay
Brazil	Japan	Peru
Cambodia	Jordan	Philippines
China	Kenya	Qatar
Colombia	Lebanon	Senegal
Cuba	Mali	Sri Lanka
Democratic Republic of the Congo	Mexico	Thailand
Dominican Republic	Mongolia	Uganda
Ecuador	Nepal	United States of America
Finland	Nicaragua	Uruguay
		Vietnam

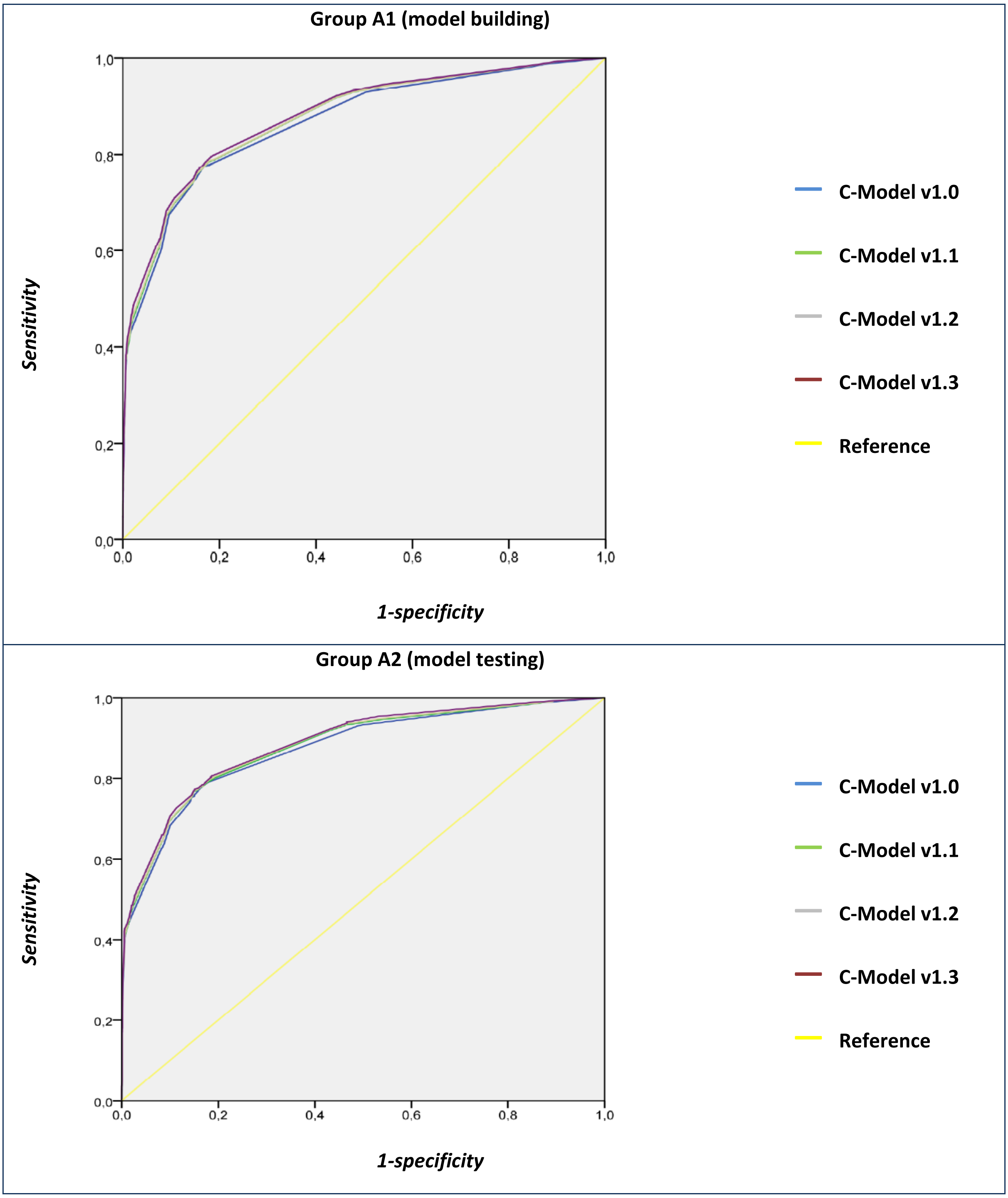

Figure S3. ROC Curves for models designed to estimate the probability of Caesarean section

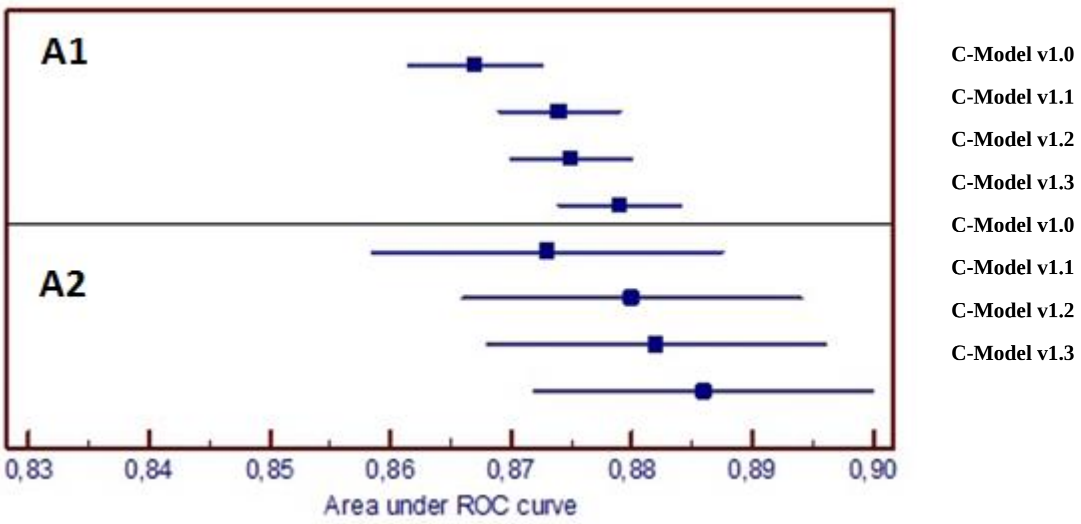

		AUC	Lower bound	Upper bound
**A1 (model building)**				
	C-Model v1.0	0·867	0·861	0·872
	C-Model v1.1	0·874	0·869	0·879
	C-Model v1.2	0·875	0·870	0·880
	C-Model v1.3	0·879	0·874	0·884
**A2 (model testing)**				
	C-Model v1.0	0·873	0·859	0·888
	C-Model v1.1	0·880	0·866	0·894
	C-Model v1.2	0·882	0·868	0·896
	C-Model v1.3	0·886	0·872	0·900

Figure S4. Forest plot of areas under the ROC curves (model building and testing)

Table S5. Classification table for assessment of the diagnostic accuracy (with 95% confidence intervals) and the percentage of cases correctly classified by models designed to estimate the probability of caesarean section*§

		Group A1 (N=38324)		Group A2 (N=4313)	
**C-Model v1.0**		Caesarean sections		Caesarean section	
		+	–	+	–
Predicted Caesarean Section	+	5245	4964	625	548
	–	1576	26539	183	2957
Sensitivity		76·89%	CI:75·88-77·88	77·35%	CI:74·34-80·10
Specificity		84·24%	CI:83·84-84·64	84·37%	CI:83·13-85·53
Diagnostic Odds Ratio		17·79	CI:16·69-18·97	18·43	CI:15·27-22·25
Percentage of correctly classified		83·0%		83·0%	
**C-Model v1.1**		Caesarean sections		Caesarean section	
		+	–	+	–
Predicted Caesarean Section	+	5252	5035	626	558
	–	1569	26468	182	2947
Sensitivity		77·00%	CI:75·98-77·98	77·48%	CI:74·47-80·22
Specificity		84·02%	CI:83·61-84·42	84·08%	CI:82·83-85·25
Diagnostic Odds Ratio		17·60	CI:16·51-18·76	18·17	CI:15·05-21·93
Percentage of correctly classified		82·8%		82·8%	
**C-Model v1.2**		Caesarean sections		Caesarean section	
		+	–	+	–
Predicted Caesarean Section	+	5333	5358	636	594
	–	1488	26145	172	2911
Sensitivity		78·19%	CI:77·19-79·15	78·71%	CI:75·76-81·40
Specificity		82·99%	CI:82·57-83·40	83·05%	CI:81·77-84·26
Diagnostic Odds Ratio		17·49	CI:16·39-18·65	18·12	CI:14·98-21·92
Percentage of correctly classified		82·2%		82·3%	

**C-Model v1.3**		Caesarean sections		Caesarean section	
		+	–	+	–
Predicted Caesarean Section	+	5233	4755	622	522
	–	1588	26748	186	2983
Sensitivity		76·72%	CI:75·70-77·71	76·98%	CI:73·95-79·75
Specificity		84·91%	CI:84·51-85·30	85·11%	CI:83·89-86·25
Diagnostic Odds Ratio		18·54	CI:17·39-19·76	19·11	CI:15·83-23·07
Percentage of correctly classified		83·5%		83·6%	

* In this analysis, any case with an estimated probability of Caesarean section above the optimal cut-off was considered a “predicted Caesarean section”. The optimal cut-off points were determined through the Youden index for each model using Group A1 ROC curves. These cut-off points were: 13·88% (Model v1.0), 9·04% (Model v1.1), 7·28% (Model v1.2), and 8·72% (Model v1.3).

§ The percentage of cases correctly classified is given by the sum of true positives and true negatives divided by all cases.

Group A1 **Group A2**

Model 1.0

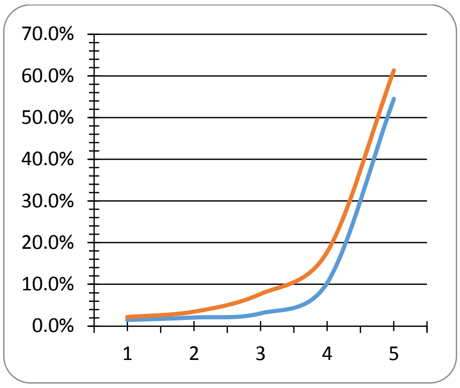

Model 1.0

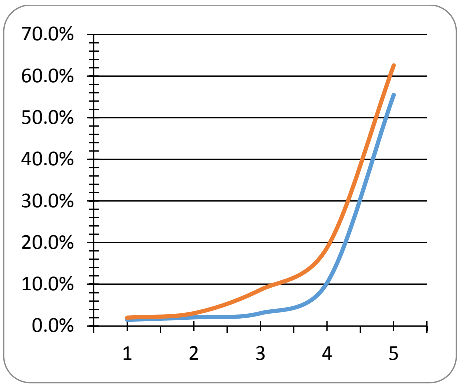

Model 1.1

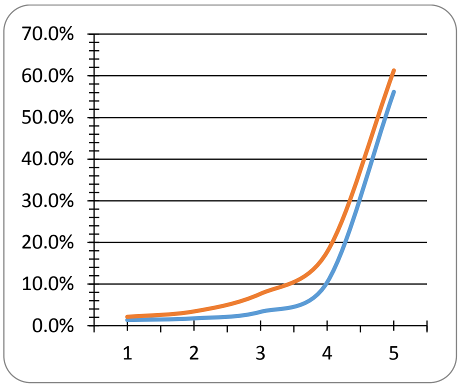

Model 1.1

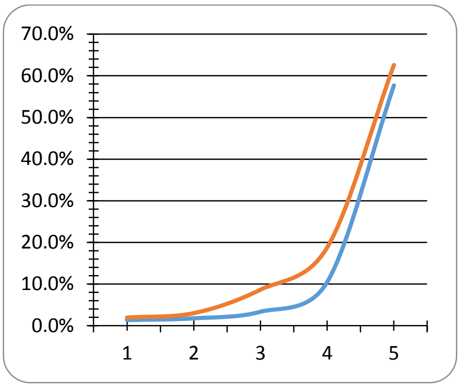

Model 1.2

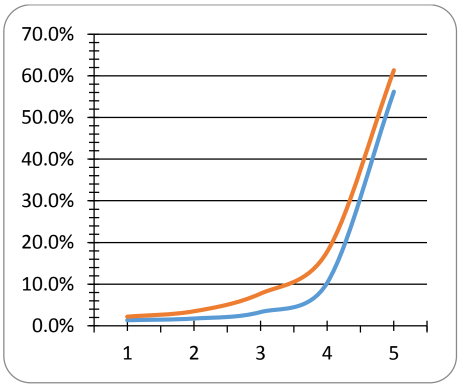

Model 1.2

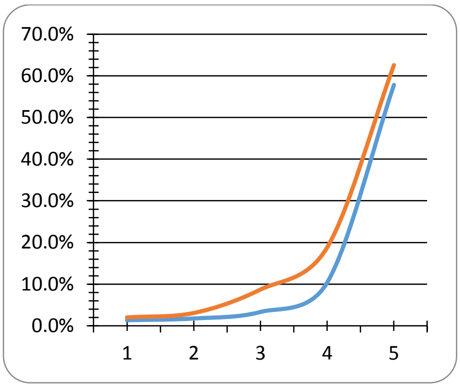

Model 1.3

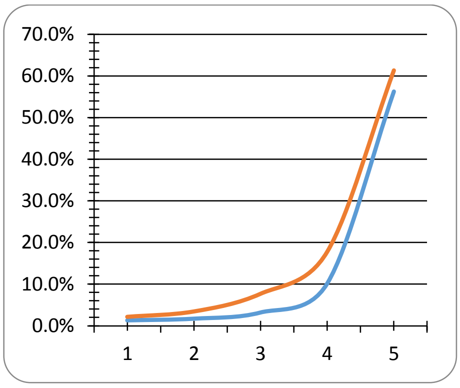

Model 1.3

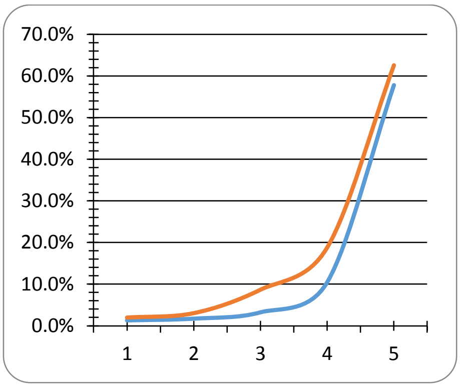

Figure S5: Predicted (—blue line) and observed (—red line) caesarean section rates by population quintiles of Caesarean section probabilities, in the Groups A1 and A2.

Table S6: Indicators of model performance

C-Model	v1.0	v1.1	v1.2	v1.3
sBrier	0.5398	0.5331	0.5309	0.5204
Pietra	0.6423	0.6499	0.6505	0.6570
Nagelkerke's R^2	0.5485	0.5559	0.5576	0.5675

Table S7. C-Model coefficients estimated using the R package*

	v1.0	v1.1	v1.2	v1.3
**Number of observations**	36853	36797	36797	36797
**Constant**	-3,34044	-3,940422	-3,937621	-3,956225
**Covariates**				
**Obstetric Characteristics**				
Parity	-0,559708	-0,759767	-0,761055	-0,776256
Previous C-section	2,841782	2,872357	2,877286	2,921585
Multiple pregnancy	1,689253	1,717075	1,715562	1,829245
Induced Labour	2,743499	2,703686	2,682078	2,629619
Presentation	2,920328	2,909807	2,92187	2,988014
Preterm birth	0,372982	0,369176	0,290626	---
**Demographics and Severity**				
Maternal Age	---	0,732938	0,726933	0,709443
Organ Dysfunction or ICU admission	---	---	1,494721	0,657152
**Complications**				
Placenta Praevia	---	---	---	2,727584
Abruptio Placentae	---	---	---	3,787302
Chronic hypertension	---	---	---	0,560569
Preeclampsia	---	---	---	0,985123
Renal disease	---	---	---	1,300909
HIV	---	---	---	1,303155

* These coefficients were calculated using the R package by an independent statistician and are very similar to those calculated using Stata (reported in the main manuscript).

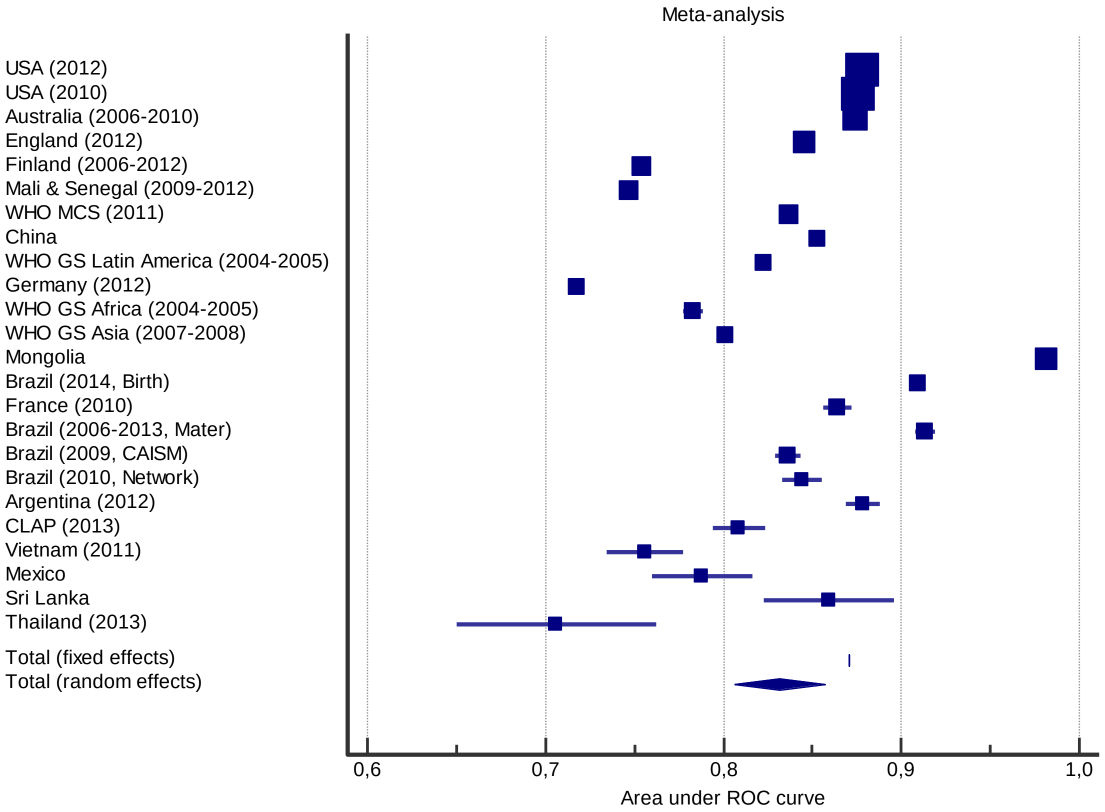

	AUC	Lower bound	Upper bound
USA (2012)	0·878	0·878	0·879
USA (2010)	0·876	0·875	0·876
Australia (2006-2010)	0·874	0·873	0·875
England (2012)	0.846	0.845	0.848
Finland (2006-2012)	0·754	0·752	0·756
Mali & Senegal (2009-2012)	0·747	0·745	0·749
WHO MCS (2011)	0·837	0·835	0·839
China	0·853	0·849	0·856
WHO GS Latin America (2004-2005)	0·823	0·82	0·826
Germany (2012)	0·718	0·715	0·722
WHO GS Africa (2004-2005)	0·783	0·778	0·789
WHO GS Asia (2007-2008)	0·801	0·798	0·804
Mongolia	0·982	0·98	0·983
Brazil (2014, Birth in Brazil)	0·909	0·905	0·912
France (2010)	0·864	0·856	0·872
Brazil (2006-2013, Mater)	0·913	0·908	0·919
Brazil (2009, CAISM)	0·836	0·829	0·843
Brazil (2010, Network)	0·844	0·833	0·855
Argentina (2012)	0·878	0·868	0·887
CLAP (2013)	0·8087	0·7937	0·8237

Vietnam (2011)	0·756	0·734	0·777
Mexico	0·788	0·759	0·816
Sri Lanka (2013-2014)	0·859	0·822	0·895
Thailand (2013)	0·706	0·650	0·762
**Summary estimate (random effects)**	0·832	0·806	0·857

Figure S6. Meta-analysis of external validation studies for the model v1.0 with 95% confidence intervals (i2=99·96%, studies ordered by the size of study population)

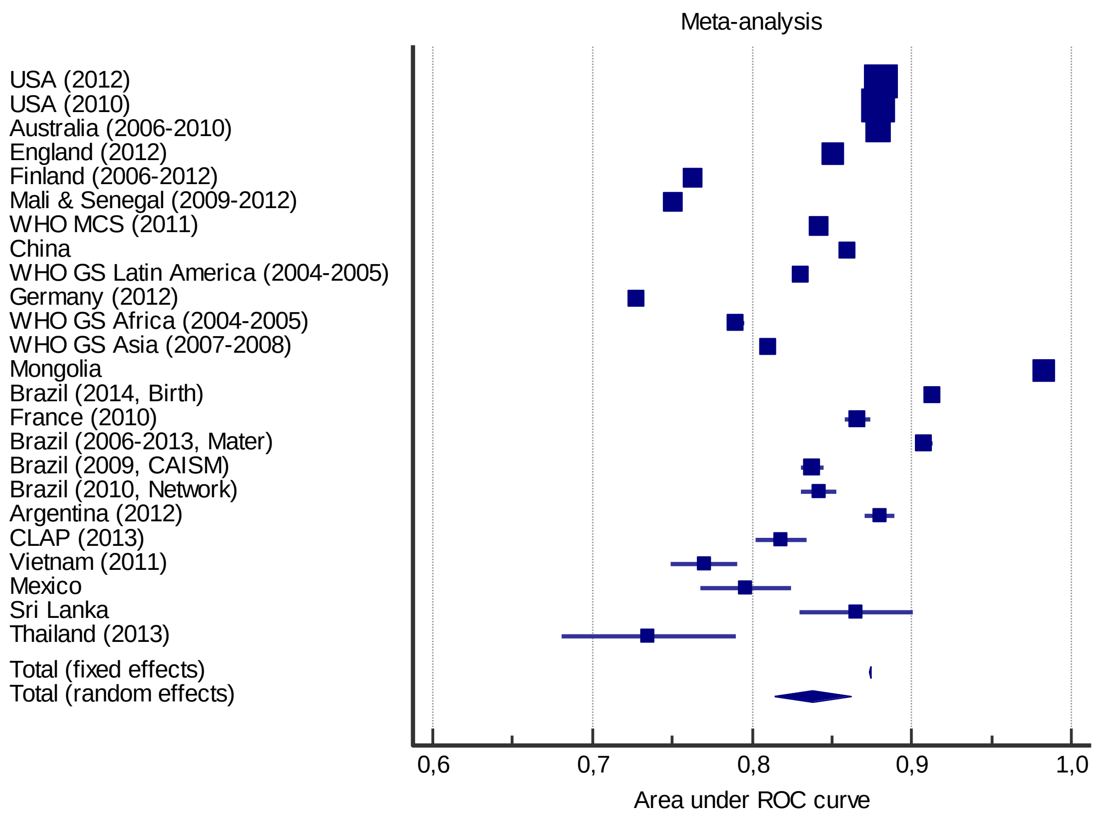

	AUC	Lower bound	Upper bound
USA (2012)	0·881	0·881	0·882
USA (2010)	0·879	0·878	0·879
Australia (2006-2010)	0·879	0·878	0·88
England (2012)	0·851	0·850	0·853
Finland (2006-2012)	0·763	0·761	0·765
Mali & Senegal (2009-2012)	0·751	0·749	0·753
WHO MCS (2011)	0·842	0·840	0·844
China	0·860	0·857	0·863
WHO GS Latin America (2004-2005)	0·831	0·828	0·834
Germany (2012)	0·728	0·724	0·731
WHO GS Africa (2004-2005)	0·790	0·785	0·795
WHO GS Asia (2007-2008)	0·810	0·807	0·813
Mongolia	0·983	0·981	0·984
Brazil (2014, Birth in Brazil)	0·913	0·909	0·917
France (2010)	0·866	0·858	0·874
Brazil (2006-2013, Mater)	0·908	0·903	0·914
Brazil (2009, CAISM)	0·838	0·831	0·845
Brazil (2010, Network)	0·842	0·831	0·853
Argentina (2012)	0·880	0·87	0·889

CLAP (2013)	0·818	0·8023	0·8342
Vietnam (2011)	0·770	0·749	0·791
Mexico	0·796	0·768	0·824
Sri Lanka (2013-2014)	0·865	0·83	0·901
Thailand (2013)	0·735	0·681	0·79
**Summary estimate (random effects)**	0·837	0·814	0·861

Figure S7. Meta-analysis of external validation studies for the model v1.1 with 95% confidence intervals (i2=99·96%, studies ordered by the size of study population)

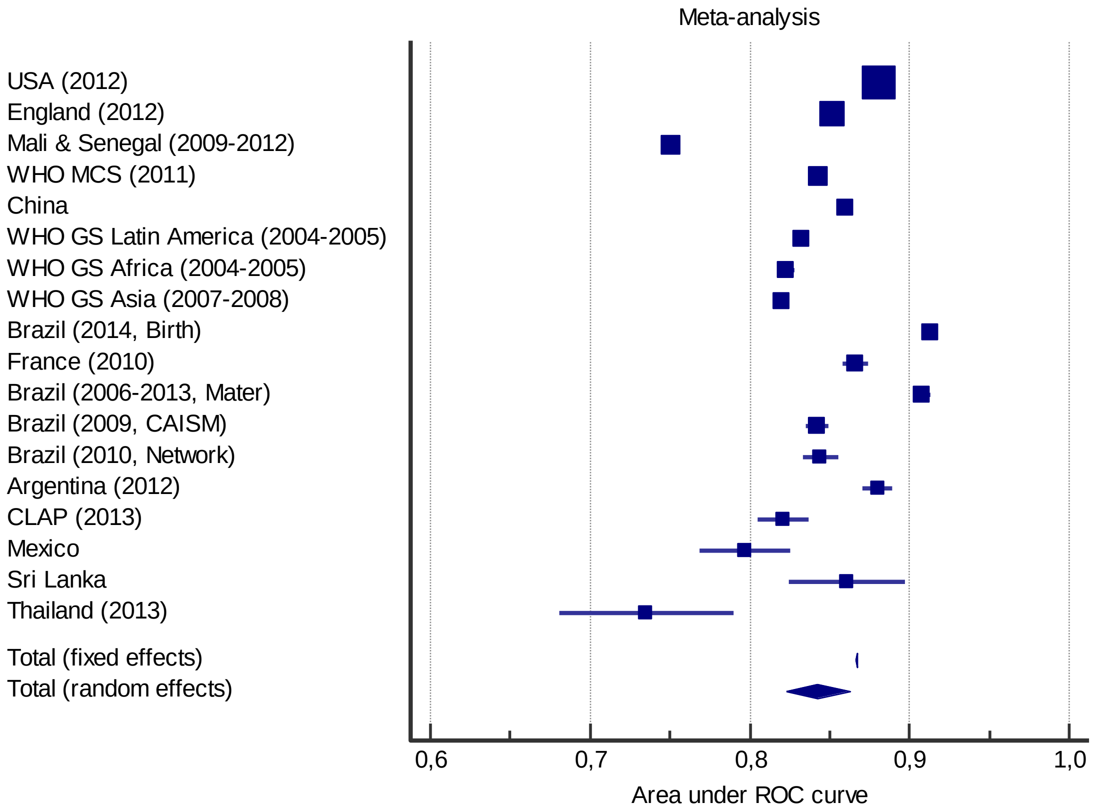

	AUC	Lower bound	Upper bound
USA (2012)	0·881	0·881	0·882
England (2012)	0·852	0·851	0·853
Mali & Senegal (2009-2012)	0·751	0·749	0·753
WHO MCS (2011)	0·843	0·841	0·845
China	0·860	0·857	0·863
WHO GS Latin America (2004-2005)	0·832	0·829	0·835
WHO GS Africa (2004-2005)	0·823	0·818	0·828
WHO GS Asia (2007-2008)	0·820	0·817	0·823
Brazil (2014, Birth in Brazil)	0·913	0·909	0·917
France (2010)	0·866	0·858	0·874
Brazil (2006-2013, Mater)	0·908	0·903	0·914
Brazil (2009, CAISM)	0·842	0·835	0·849
Brazil (2010, Network)	0·844	0·833	0·855
Argentina (2012)	0·880	0·871	0·890
CLAP (2013)	0·821	0·805	0·836
Mexico	0·797	0·768	0·825
Sri Lanka (2013-2014)	0·861	0·825	0·898
Thailand (2013)	0·735	0·681	0·790
**Summary estimate (random effects)**	0·842	0·823	0·862

Figure S8. Meta-analysis of external validation studies for the model v1.2 with 95% confidence intervals (i2=99·92%, studies ordered by the size of study population)

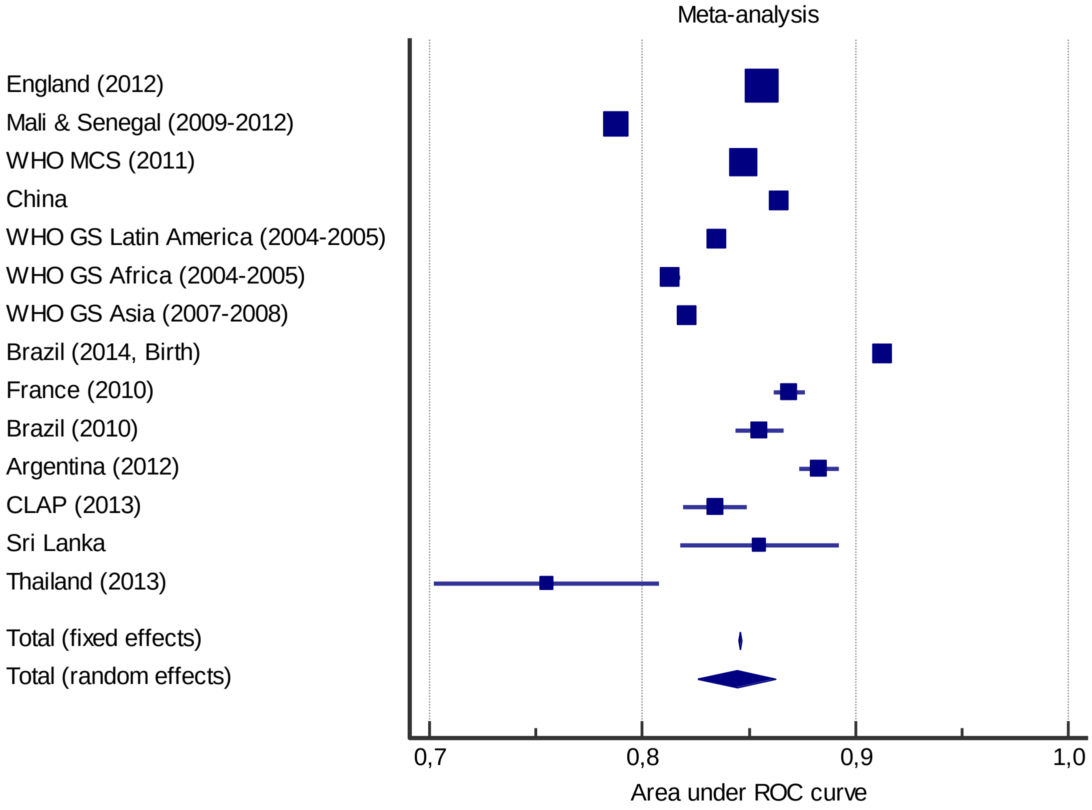

	AUC	Lower bound	Upper bound
England (2012)	0·856	0·855	0·857
Mali & Senegal (2009-2012)	0·788	0·786	0·79
WHO MCS (2011)	0·848	0·846	0·849
China	0·864	0·861	0·867
WHO GS Latin America (2004-2005)	0·835	0·832	0·838
WHO GS Africa (2004-2005)	0·813	0·808	0·817
WHO GS Asia (2007-2008)	0·821	0·818	0·824
Brazil (2014, Birth in Brazil)	0·913	0·909	0·916
France (2010)	0·869	0·862	0·877
Brazil (2010)	0·855	0·844	0·866
Argentina (2012)	0·883	0·873	0·892
CLAP (2013)	0·8341	0·819	0·8492
Sri Lanka (2013-2014)	0·855	0·818	0·893
Thailand (2013)	0·755	0·702	0·808
**Summary estimate (random effects)**	0·844	0·826	0·863

Figure S9. Meta-analysis of external validation studies for the model v1.3 with 95% confidence intervals (i2=99·78%)

Table S8. Sensitivity analysis including only databases with complete data for all C-Model versions. (summary estimates of areas under the ROC curves with 95% confidence intervals; random effects meta-analyses).

	AUC	Lower bound	Upper bound
Sensitivity Analysis			
C-Model v1.0*	0·828	0·803	0·853
C-Model v1.1*	0·834	0·811	0·857
C-Model v1.2*	0·838	0·816	0·860
C-Model v1.3*	0·844	0·826	0·863

Table S9: Sensitivity

Country	Brief description
Argentina	
Australia	**National Perinatal Data Collection (NPDC)**<br>The National Perinatal Data Collection (NPDC) is a national population-based cross sectional data collection of pregnancy and childbirth. The data are based on births reported to the perinatal data collection in each state and territory in Australia. Midwives and other staff, using information obtained from mothers and from hospital or other records, complete notification forms for each birth. Information is included in the NPDC on both live births and stillbirths of at least 400 grams birthweight or at least 20 weeks gestation. The NPDC is compiled annually by the National Perinatal Epidemiology and Statistics Unit.<br>https://npesu.unsw.edu.au/data-collection/national-perinatal-data-collection-npdc
Brazil - CAISM	UNICAMP WOMEN'S HOSPITAL (CAISM) - INTRAHOSPITAL INFORMATION SYSTEM
Brazil - SAMM	**Brazilian network for the surveillance of maternal potentially life threatening morbidity and maternal near-miss and a multidimensional evaluation of their long term consequences**<br><br>This network implemented a multicenter, cross-sectional study in 27 referral obstetric units in different geographical regions of Brazil. Over 12 months, investigators performed prospective surveillance to identify all maternal complications. The study population comprised all women surviving potentially life-threatening conditions (severe maternal complications) or life-threatening conditions (the maternal near miss criteria) and maternal deaths according to the new WHO definition and criteria.<br><br>Cecatti JG, Souza JP, Parpinelli MA, Haddad SM, Camargo RS, Pacagnella RC,Silveira C, Zanardi DT, Costa ML, Pinto e Silva JL, Passini R Jr, Surita FG, Sousa MH, Calderon IM, Say L, Pattinson RC; Brazilian Network for Surveillance of Severe Maternal Morbidity. Brazilian network for the surveillance of maternal potentially life threatening morbidity and maternal near-miss and a multidimensional evaluation of their long term consequences. Reprod Health. 2009 Sep 24;6:15. doi: 10.1186/1742-4755-6-15
Brazil - Mater	CENTRO DE REFERENCIA DE SAUDE DA MULHER DE RIBEIRAO PRETO (MATER) - INTRAHOSPITAL INFORMATION SYSTEM
Brazil – Birth in Brazil	Database of the Birth in Brazil Project, which collected perinatal information in 266 randomly selected health facilities, with national representativeness.
CLAP	LATIN AMERICA STUDY ON SEVERE MATERNAL MORBIDITY (CLAP)

England	Information on the Hospital Episodes Statistics: HES is a data 'warehouse' that includes records of all inpatient admissions and day cases in English NHS trusts, with the data being extracted from local patient administration systems. In HES, each record contains data on the patient demographics (for example, age, sex, ethnicity, postcode), the episode of care (for example, hospital name, date of admission and discharge) and clinical information. Diagnoses for each patient are recorded using the International Classification of Diseases, 10th edition (ICD-10) (WHO, 2010). Procedures performed during an episode are coded using the Office of Population, Censuses and Surveys Classification of Surgical Operations and Procedures, 4th revision (OPCS) (Health and Social Care Information Centre, 2014). In addition, each episode related to the delivery of a baby can capture details about the labour and delivery (for example, parity, mode of delivery, gestational age, birthweight) in supplementary data fields known as the HES 'maternity tail'. Over 96% of all deliveries in England occur in NHS hospitals and are therefore captured by HES (Birthplace in England Collaborative Group, 2011).<br><br>1) Birthplace in England Collaborative Group (2011) Perinatal and maternal outcomes by planned place of birth for healthy women with low risk pregnancies: the Birthplace in England national prospective cohort study. BMJ 343:d7400<br><br>2) Health and Social Care Information Centre (2014) Office of Population, Censuses and Surveys Classification http://systems.hscic.gov.uk/data/clinicalcoding/codingstandards/opcs4<br><br>3) Bragg F, Cromwell DA, Edozien LC, Gurol-Urganci I, Mahmood TA, Templeton A, van der Meulen JH. Variation in rates of caesarean section among English NHS trusts after accounting for maternal and clinical risk: cross sectional study. BMJ 2010;341:c5065. doi: 10.1136/bmj.c506
Finland	Finnish National Database.<br>THL National Institute for Health and Welfare, Helsinki, Finland and NHV Nordic School of Public Health, Gothenburg, Sweden.
France	2010 French National Perinatal Survey - The French Perinatal Surveys[1] are repeated cross-sectional surveys based on representative samples of births in France. Their aims are to monitor health status and perinatal care and to assess medical practices and programs. Data collection covers all births during one week, that is, all live born or stillborn children, in all French public and private maternity units, as well as children born outside these institutions and subsequently transferred to one, at a gestational age of at least 22 weeks or weighing at least 500 grams. The 2010 survey included 14681 women and 14898 children. The information came from an interview with the women in postpartum ward about social and demographic characteristics and prenatal care, and from medical files for the management and complications of pregnancy and delivery and the newborn’s health status. Inserm coordinated the study at national level, and data were collected by trained midwives.<br>Blondel B., Lelong N., Kermarrec M., Goffinet F., the National Coordination Group of the National Perinatal Surveys. *Trends in perinatal health in France from 1995 to 2010. Results from the French National Perinatal Surveys.* J Gynecol Obstet Biol Reprod (Paris) 2012. 41(4): p. e1-e15.
Germany	The German data are taken from a standard nationwide perinatal data set defined solely for the purposes of monitoring quality of care in German hospitals. For the present analysis the data were restricted to the state of Bavaria, constituting approximately 14% of all German births. The Bavarian data may be considered as representative for the entire German data with little regional variation. The data are not registry data compiled for the sake of generating official national reports. However, the data checks are rigorous and the perinatal data set has remained stable for more than 10 years. The quality of care in German hospitals is reported annually for many other fields of care apart from obstetric care.

Mali & Senegal	Our perinatal database was developed for the QUARITE Trial in 46 referral hospitals in Mali and Senagal. QUARITE is an international, multi-centre, controlled cluster-randomized trial of a complex intervention. Inclusion criteria for hospital are: functional operating rooms and more than 800 deliveries annually. Exclusion criteria are: private health care facility, already had a structured program for carrying out maternal death audits, written consent not provided by local authorities. Inclusion criteria for women in the QUARITE study are 1) being a patient who delivered in one of the participating facilities, 2) between September 1, 2007, and August 31, 2011. Exclusion criteria are 1) having delivered at home or 2) in another centre, with postnatal transfer. The database is based on the WHO global survey on maternal and perinatal health, which considers clinical data at the individual level and organizational data at the facility level. All deliveries carried out in the participating centres are registered by local collectors (nurses or midwives trained to do this). These collectors complete a standard form for each eligible patient that includes information on maternal characteristics, prenatal care, labour and delivery, diagnosed complications, and the vital status of both mother and child at discharge from hospital.<br><br>Dumont A, Fournier P, Abrahamowicz M, Traoré M, Haddad S, Fraser W. QUARITE (quality of care, risk management, and technology in obstetrics): a cluster-randomized trial of a multifaceted intervention to reduce hospital-based maternal mortality in Senegal and Mali. Lancet 2013; 382: 9887, 146–57. Published online May 28, 2013 http://dx.doi.org/10.1016/S0140-6736(13)60593-0
Mexico	Data are from a study aimed at evaluating the quality of basic obstetric care provided by general physicians, obstetric nurses and professional midwives in Mexico (1). Data collection was conducted between 2006 and 2007 in 5 hospitals with these provider types. Data was collected by direct observation of deliveries and review of medical records of women delivering at participating hospitals. Data collection was focused on evidence-based recommended obstetric care at admission, active phase of delivery and postpartum. All but one hospital had the capacity to perform c-section 24 hours a day. Information was available for all variables of C-model, except chronic hypertension. Data was insufficient to completely differentiate among breech and other non-cephalic presentations. Preterm birth was recorded as gestational age <36 weeks. Data on admission to ICU was not available. Organ dysfunction was defined as the presence of any of the following: heart failure, cardiac arrest, hemorrhagic/septic shock, disseminated intravascular coagulation, intraventricular hemorrhage, hysterectomy, and maternal death. The study that generated the data used in this validation included women admitted in labor. Thus women with an absolute indication for c-section were excluded. While the c-section rate in this study was 27%, in 2012, the Mexican c-section rate was 55% (1). The dataset from this study can be used for applying the C-model and performing ROC analysis. However, given that this is a dataset with unique characteristics, results from this validation should only be extrapolated to the facility-based care for the population of women already presenting in labor in Mexico.<br><br>1) Walker D, DeMaria LM, Suarez L, Cragin L, and The Evaluating Alternative Models of Obstetric Care in Mexico Research Team. Skilled birth attendants in Mexico: how does care during normal birth by general physicians, obstetric nurses, and professional midwives compare with World Health Organization evidence-based practice guidelines? J Midwifery Womens Health 2012;57:18–27.<br><br>2) Suárez-López L, Campero L, De la Vara-Salazar E, Rivera-Rivera L, Hernández-Serrato MI, Walker D, Lazcano-Ponce E. [Sociodemographic and reproductive characteristics associated with the increase of cesarean section practice in Mexico]. Salud Publica Mex 2013;55 suppl 2:S225-S234.
Mongolia	Maternity hospitals' data of the childbirth 2011 was used for the analysis. All deliveries took in 3 maternity hospitals in the capital city and The National Center for Maternal and Child Health were collected and managed by the Ministry of Health, Mongolia. It covered all births in the Ulaanbaatar plus all high risk deliveries referred to the tertiary level from all provinces.

Sri Lanka	For the C- model validation study, we selected the premier women’s hospital with a higher average number of deliveries per year in Sri Lanka. Our aim was to have a random sample of women from this hospital to represent 5% of annual admissions. Based on the available records, around 9388 deliveries take place in this hospital annually. We randomly selected 496 deliveries (5.3% of total deliveries) taken place in the months of December 2013 and January 2014. Three MBBS qualified medical officers manually extracted all variables required for the validation from paper based patient records available in record room of the hospital. There were no maternal deaths or pregnant women with HIV infections admitted to the hospital during the study period.
Thailand	In Thailand, the C model was tested in a sample of 300 deliveries randomly selected from the medical record deliveries of Kalasin hospital during September-December 2013. Kalasin hospital is a regional hospital in the northeast region. The hospital provides 40 beds for obstetric care and has an average of 3700 deliveries annually. During the four months, caesarean section rate was 52.3 %. The data was kindly supported by Dr. Bunpode Suwannachart, the vice director of Kalasin regional hospital. His email address is bunpode@yahoo.com
USA 2010	The 2010 US Natality Public Use File contains all registered live births that occurred in the Ò50 states, the District of Columbia, and New York CityÓ in 2010 . We restricted our analysis to data from 35 states , the District of Columbia, and New York City since these locations had adopted the 2003 revision of the U.S. Standard Certificate of Live Birth by January 1, 2010. Some Louisiana and North Carolina births are not included since these states adopted the 2003 birth certificate after January 1. We excluded births with missing values for the explanatory variables, implausible values for the number of previous cesarean sections, or a total birth order ³8 . After exclusions, there were 2,892,041 live births included in the analysis of C-models 1 and 2. National Center for Health Statistics. User Guide to the 2010 Natality Public Use File. Hyattsville, Maryland: National Center for Health Statistics. Annual product 2012. Available for downloading at: http://www.cdc.gov/nchs/data_access/VitalStatsOnline.htm.<br>California, Colorado, Delaware, Florida, Georgia, Idaho, Illinois, Indiana, Iowa, Kansas, Kentucky, Louisiana, Maryland, Michigan, Missouri, Montana, Nebraska, Nevada, New Hampshire, New Mexico, New York, North Carolina, North Dakota, Ohio, Oklahoma, Oregon, Pennsylvania, South Carolina, South Dakota, Tennessee, Texas, Utah, Vermont, Washington, and Wyoming.<br>In 2010, there were 3,101,037 recorded births in states/cities that used the 2003 revision of the live birth certificate; these were eligible for inclusion in our analysis. After exclusions, 2,892,041 live births were included in the analysis of C-models 1 and 2.<br>Since women with a total birth order of 8 or more were combined in the US natality file, it was not possible to determine whether these women had implausible values for number of previous cesarean sections. Hence, they were excluded.

Goldenberg RL, McClure EM, Bann CM. The relationship of intrapartum and antepartum stillbirth rates to measures of obstetric care in developed and developing countries. Acta Obstet Gynecol Scand 2007;86(11):1303-9.

USA 2012	The 2012 US Natality Public Use File contains all registered live births that occurred in the Ò50 states, the District of Columbia, and New York CityÓ in 2012. We restricted our analysis to data from 39 states , the District of Columbia, and New York City since these locations had adopted the 2003 revision of the U.S. Standard Certificate of Live Birth by January 1, 2012. Some Virginia births are not included since Virginia adopted the 2003 birth certificate after January 1. We excluded births with missing values for the explanatory variables, implausible values for the number of previous cesarean sections, or a total birth order ³8 . After exclusions, there were 3,283,819 live births included in C-models 1 and 2 and 3,279,483 live births included in C-model 3. National Center for Health Statistics. User Guide to the 2012 Natality Public Use File. Hyattsville, Maryland: National Center for Health Statistics. Annual product 2014. Available for downloading at: http://www.cdc.gov/nchs/data_access/VitalStatsOnline.htm.<br>The 39 states were California, Colorado, Delaware, Florida, Georgia, Idaho, Illinois, Indiana, Iowa, Kansas, Kentucky, Louisiana, Maryland, Massachusetts, Michigan, Minnesota, Missouri, Montana, Nebraska, Nevada, New Hampshire, New Mexico, New York, North Carolina, North Dakota, Ohio, Oklahoma, Oregon, Pennsylvania, South Carolina, South Dakota, Tennessee, Texas, Utah, Vermont, Virginia, Washington, Wisconsin and Wyoming.<br>In 2012, there were 3,495,710 recorded births in states/cities that used the 2003 revision of the live birth certificate; these were eligible for inclusion in our analysis. After exclusions, 3,283,819 live births were included in C-models 1 and 2, and 3,279,483 were included in C-model 3.<br>Since women with a total birth order of 8 or more were combined in the US natality file, it was not possible to determine whether these women had implausible values for number of previous cesarean sections. Hence, they were excluded.<br><br>Zhang J, Troendle J, Reddy UM, et al. Contemporary cesarean delivery practice in the United States. Am J Obstet Gynecol 2010;203:326.e1-10
Vietnam	Vietnam data - The prospective facility based study included 3056 women who live in the catchment area of one city in Vietnam including eight community health centres and one provincial hospital in 2007-2008.<br><br>Ota E, Haruna M, Suzuki M, et al. Maternal body mass index and gestational weight gain and their association with perinatal outcomes in Viet Nam. Bulletin of the World Health Organization 2011; 89(2), 127-136.
WHO GS Asia	Souza JP, Gülmezoglu A, Lumbiganon P, et al. Caesarean section without medical indications is associated with an increased risk of adverse short-term maternal outcomes: the 2004-2008 WHO Global Survey on Maternal and Perinatal Health. BMC Med. 2010; 10;8:71.
WHO MCS	Souza JP, Gülmezoglu AM, Vogel J, Carroli G, Lumbiganon P, Qureshi Z, et al. Moving beyond essential interventions for reduction of maternal mortality (the WHO Multicountry Survey on Maternal and Newborn Health): a cross-sectional study. Lancet 2013; 18;381(9879):1747-55.
WHO GS LA	Souza JP, Gülmezoglu A, Lumbiganon P, et al. Caesarean section without medical indications is associated with an increased risk of adverse short-term maternal outcomes: the 2004-2008 WHO Global Survey on Maternal and Perinatal Health. BMC Med 2010; 10;8:71.

WHO GS Africa	Souza JP, Gülmezoglu A, Lumbiganon P, et al. Caesarean section without medical indications is associated with an increased risk of adverse short-term maternal outcomes: the 2004-2008 WHO Global Survey on Maternal and Perinatal Health. BMC Med 2010;8:71.

C-Model calculator

C-model's online website was developed using the CakePHP framework 1, a responsive layout template based on Bootstrap 2, called AdminLTE 3. The database management system adopted was MySql4. This feature contains two main functionalities and the first one is the calculator, in which it is possible to calculate promptly the individual probability of C-Section by choosing the available options. The second one is a tool for database analysis, in which a user can submit a .csv or .xls file, following the system pattern, and in return, receive the Observed, Expected and Standardized C-Section Rates, the Uncertainty Range and the ROC Curve for the submitted file.

C-model calculator was developed by: Livia Oliveira-Ciabati, Alexandre Freitas Duarte, Newton Shydeo Brandão Miyoshi
